# On the Feasibility of an LCD-Based Real-Time Converter for Ionizing Radiation Imaging

**DOI:** 10.3390/ma17133320

**Published:** 2024-07-04

**Authors:** Adam Januszko, Eugeniusz Zych, Wiktor Piecek, Witalis Pellowski, Krzysztof A. Bogdanowicz, Agnieszka Iwan

**Affiliations:** 1 Faculty of Security and Safety Research, General Tadeusz Kosciuszko Military University of Land Forces, Czajkowskiego 109, 51-147 Wroclaw, Poland; witalis.pellowski@awl.edu.pl; 2 Faculty of Chemistry, University of Wroclaw, 14 F. Joliot-Curie Street, 50-383 Wroclaw, Poland; eugeniusz.zych@uwr.edu.pl; 3 Faculty of Advanced Technologies and Chemistry, Military University of Technology, 00-908 Warsaw, Poland; wiktor.piecek@wat.edu.pl; 4 Military Institute of Engineer Technology, Obornicka 136, 50-961 Wroclaw, Poland; bogdanowicz@witi.wroc.pl

**Keywords:** converter of X and gamma radiation, liquid crystal displays, twisted nematic, security engineering

## Abstract

Here we present the cascade converter (CC), which provides real-time imaging of ionizing radiation (IoR) distribution. It was designed and manufactured with the simplest architecture, utilizing liquid crystal display (LCD) technology. Based on two merged substrates with transparent electrodes, armed with functional layers, with the cell filled with nematic liquid crystal, a display-like, IoR-stimulated CC was achieved. The CC comprises low-absorbing polymer substrates (made of polyethylene terephthalate—PET) armed with a transparent ITO electrode covered with a thin semipermeable membrane of polymer (biphenylperfluorocyclobutyl: BP-PFCB) doped with functional nanoparticles (NPs) of Lu_2_O_3_:Eu. This stack was covered with a photoconductive layer of α-Se and finally with a thin polyimide (PI) layer for liquid crystal alignment. The opposite substrate was made of LCD-type glass with ITO and polyimide aligning layers. Both substrates form a cell with a twisted structure of nematic liquid crystal (TN) driven with an effective electric field $E_{eff}$. An effective electric field driving TN structure is generated with a sum of (1) a bias voltage $V_{BIAS}$ applied to ITO transparent electrodes and (2) the photogenerated additional voltage $V_{Xray}$ induced between ITO and α-Se layers with a NPs-doped BP-PFCB polymer layer in-between. The IoR (here, X-ray) conversion into real imaging of the IoR distribution was achieved in the following stages: (1) conversion of IoR distribution into non-ionizing red light emitted with functional NPs, (2) transformation of red light into an electric charge distributed in a layer of the photoconductive α-Se, which is what results in the generation of distributed voltage $V_{Xray}$, and (3) a voltage-mediated, distributed switching of the TN structure observed with the naked eye. The presented imaging device is characterized by a simple structure and a simple manufacturing process, with the potential for use as a portable element of IoR detection and as a dosimeter.

## 1. Introduction

Efficient and cheap imaging technologies for X-ray and/or gamma radiation (denoted as ionizing radiation—IoR) detection and visualization have been searched for decades. Current solutions are based on materials that change their resistivity while illuminated with IoR [1,2,3,4,5]. Several versions of IoR converters have been proposed up to date. It is worth noting that devices with a limited number of electronic components are based on the cascade conversion of IoR, inducing a distribution of charge (or conductivity), followed by a respective distribution of emission or transmission of visible light, thus providing the final imaging of IoR.

For instance, the device described in the US patent [1] converts the IoR incident on a photoconductive panel into an image that can be directly observed. The device consists of a thick plate of photoconductive material, made of bismuth oxide or a mixture of bismuth oxide with silicon or germanium oxide, which exhibits very high resistivity in the absence of radiation and high photoconductivity when illuminated with IoR. The device incorporates a nematic liquid crystal (LC) layer placed between two polarizers which allows the image to be produced as a response to the IoR-mediated distribution of the voltage. 

Paper [2] discloses a device for X-ray intensity imaging that includes a sensing layer absorbing X-ray radiation, an image-forming layer, and an amplifier stage. The device is based on a photoconductive X-ray detector that due to the IoR-generated charge distribution creates an image into an LC image-forming layer. The detector contains twisted nematic liquid crystal cells (TN) placed between two crossed polarizers, mounted over a photoconductive layer of amorphous selenium. The LC image-forming layer changes the intensity of the light transmitted through the entire structure. A CCD camera records an optical image and transmits it to the image processor where it is digitally processed and then displayed for the readout. The whole system, except the image recording layer containing liquid crystal cells with a selenium layer, requires an additional light source and CCD cameras for recording. This fact determines the lack of compactness of the device and increases its production and operating costs. 

In turn, the patent [3] introduces a digital system for X-ray diagnostics. The device is composed of a photoconductive detector and an electro-optical modulator. The photoconductive detector absorbs X-rays that have previously passed through the object being tested, creating an image of the object that is stored in an electro-optical modulator. In the cited patent [3], the photoconductive detector layer is amorphous selenium, which is adjacent to the electro-optical modulator based on liquid crystal. The X-ray image created in this manner is stable for several minutes and can be digitized using scanning techniques or a CCD camera. The device mentioned above records static images only, not allowing for the live view. Furthermore, recording a subsequent image necessitates an erasing procedure that employs a beam of visible light within a specific range of wavelengths. A subsequent patent [4] presents a device generating X-ray-mediated visualization containing an X-ray source and an X-ray detector. The detector consists of a photoconductive layer of amorphous selenium with a thickness of 50–500 µm and an LC-based electro-optical modulator. Additionally, the device includes a non-actinic (not exposing the photoconductive layer) light source to create an optical representation of the exposed X-ray image, an image sensor that collects the projected image using non-actinic light, and a processor connected to the image sensor to extract, process, and store images. The mentioned device contains several complex elements, such as the second IoR source, image converter or processor which make the device complicated and expensive to produce and operate. 

In the patent [5], a flat, thin-film detection panel is used in the form of a pixel matrix, serving as a real-time imaging device and a dosimeter for X-ray or gamma-ray detection. It includes a series of photodiodes made of hydrogenated amorphous silicon sited on a glass substrate. The detection core of the aforementioned device is a layer that converts X-ray or gamma radiation into an electric field, consisting of a selenium layer with a thickness of 300–500 µm. The electric field generated in the conversion layer then causes the Thin-Film Transistor (TFT) of the corresponding pixel to switch, thereby creating an image. The use of a selenium layer with a thickness of up to 500 μm is a technological challenge. Moreover, it additionally reduces the transparency of the entire device, limiting the detection sensitivity. What is more, the application process is that a thick layer of selenium can recrystallize, eventually generating a dark current.

In addition to the aforementioned common devices for IoR detection, visualization, and dosimetry [6], new ideas for devising them have appeared. They are based on wide bandgap materials such as perovskites (manifesting a superior sensitivity and stability) [7,8] or diamond which is the most promising material for X-rays and high-energy particle detection [9,10,11]. 

The cascade converter (CC) discussed in this paper is based on the patent [12] describing a dual-use IoR detector for military and civil applications. The military application of the discussed IoR converter is a dosimeter with a direct, real-time equivalent dose reading. A simple, compact design allows for the implementation of a CC, e.g., in the eyepiece of a gas mask or on the screen of any display, glasses, binoculars, periscope, and as an additional display/indicator of the IoR. The main goal of this work is to describe the design of a simplified IoR converter and an X-ray imaging device based on smart materials and structures, characterized by the following properties:

(a) using a minimum of components, which makes the CC simpler than the devices mentioned above,
(b) real-time imaging of the IoR intensity distribution and reading with the naked eye,
(c) design of the CC allowing for working in transmission and/or reflection mode,
(d) final readout operating with a visible light,
(e) increased sensitivity for IoR detection,
(f) low power consumption.

The designed LCD-based converter allows for direct observation of the grayscale picture resulting from the IoR-mediated electro-optical effect in the LC structure.

## 2. Ionizing Radiation

### 2.1. Radiation Detectors and Active and Passive Radiation Imaging

**Figure 1:**
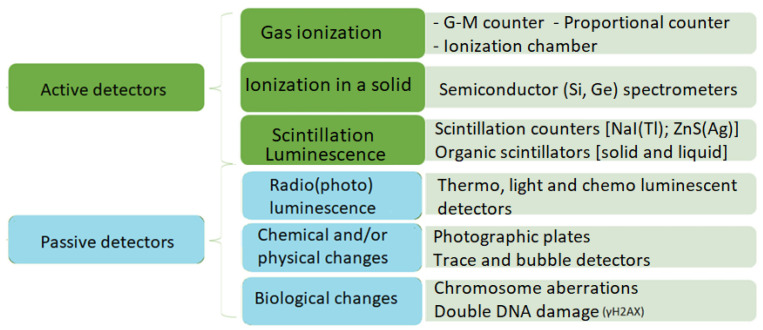
General classification of IoR detectors.

Ionizing radiation (IoR) is radiation of energy allowing for the ionization of atoms or molecules, usually leading to the creation of electric charges, like electron–hole pair, excitation of atoms or molecules, and space-resolved charge distribution. Authors of analyses in this field adopt different division criteria and classify detectors in various ways. In this paper, we propose simplified classification criteria [13] for active and passive detectors as depicted in Figure 1.

Active detectors require power supply and measurement systems, while passive ones allow for visual imaging of physical and/or chemical changes in the detector volume (sometimes separate reading devices are also necessary). 

Many tools and measurement instruments are used in radiometric practice—using various physical and chemical phenomena. Generally, radiometric instruments can be divided into active and passive. Active methods use the phenomenon of interaction of radiation with the material (gas, liquid, solid) and devices that record these changes. In passive methods, the presence of radiation is observed online or after technological processing. For example, ionization chambers are used in many fields such as medicine [14,15], radiation protection [16,17], industry [18,19], nuclear energy [20,21], and scientific research [22,23] due to their ability to precisely measure ionizing radiation. In radiotherapy, the ionization chambers are used to calibrate and monitor radiation doses in cancer therapy [14], while in diagnostic imaging they are used to calibrate X-ray and CT scanners [15]. Moreover, these are also used to measure radiation levels in public places and industrial plants [16], in personal dosimeters for workers exposed to radiation [17], in the food and pharmaceutical industries to detect contamination [18], to test materials in industry (non-destructive testing) [19], to measure radiation levels in nuclear reactors [20], to monitor waste before it is stored [21], in nuclear and particle physics experiments [22], and in studies of cosmic rays [23].

Proportional counters are radiation detectors that use the ionization phenomenon to detect and measure ionizing radiation such as nuclear and particle physics and X-ray and gamma-ray spectroscopy [22,24,25,26], radiation protection [16,27], industry [28,29], geology and archeology [30,31], or medicine [32,33]. For example, the proportional counters are used to analyze the chemical composition of materials by detecting characteristic X-rays [24,25], in nuclear physics experiments to detect and measure the energy of alpha, beta and neutron particles [22,26], to measure radiation levels in the environment to ensure compliance with radiation safety standards [16,27], for quality control in the pharmaceutical, food, and materials industries by analyzing the elemental composition [28,29], to date geological and archaeological samples by measuring their radioisotope content [30,31], or in medical diagnosis to measure radiation in diagnostic procedures such as X-rays [32,33]. 

Well-known Geiger–Müller (G–M) counters are versatile ionizing radiation detectors that are widely used in many fields of science and industry. For example, Geiger–Müller counters are used to detect and measure radiation levels in the environment to ensure compliance with radiation safety standards [16], to monitor radiation levels in industrial processes, for example, in the production of radioactive materials [34], for radiation detection in non-destructive testing such as weld inspection [19], to detect radiation in medical diagnostics, e.g., in radioisotope studies [33], to monitor and calibrate radiation sources used in cancer therapy [35], to monitor cosmic radiation aboard satellites and space stations [23], or to detect deposits of uranium and other radioactive elements in the earth [33], and many others.

Gamma-ray spectrometers are advanced devices used to analyze gamma-ray spectra, enabling the identification and quantitative measurement of various radioisotopes. For example, they are used to monitor radioactive activity in nuclear reactors and to analyze the composition of nuclear fuel [24], to analyze and classify radioactive waste before disposal [36], to measure and analyze radiation levels in the natural environment, including air, water, and soil [16], in radiological emergency situations to identify and monitor radiation sources [37], in the study of nuclear structure and nuclear reactions [38], to analyze the elemental composition of materials in various industries, including chemical and metallurgical industries [39], to determine the age of geological and archaeological samples by analyzing the content of radioisotopes [31], to detect deposits of uranium and other radioactive elements [40], in medical diagnostics to identify and measure isotopes used in nuclear medicine [32], or to calibrate and monitor radiation sources used in cancer therapy [35]. 

Also, radiation luminescence (scintillation) methods are widely used in various fields of science and industry due to their ability to detect and measure ionizing radiation through the emission of generated light. Scintillators are used in single photon emission computed tomography (SPECT) and positron emission tomography (PET) to image metabolic processes in the body [41], to monitor the doses of radiation delivered to patients undergoing cancer treatment [42], are key components in detectors used in high-energy physics experiments such as those at CERN [43], in cosmic ray detectors aboard satellites and space probes [23], to measure radiation levels in the environment, both in public places and industrial facilities [16], to control the quality of industrial products, e.g., in the food and pharmaceutical industries [39], in non-destructive testing to detect defects in materials [19], in radiometric dating and analysis of geological samples [31], in scintillation spectroscopy to analyze the chemical composition of samples [27], to monitor radiation levels in nuclear reactors and to analyze the composition of nuclear fuel [20], and to analyze and classify radioactive waste before disposal [36]. 

Chemical radiation detectors, which use chemical changes to detect ionizing radiation, have a wide range of applications in various fields. For example, they are used to monitor radiation levels in the natural environment and in public and industrial places [16], to monitor radiation doses in diagnostic and therapeutic procedures, including radiotherapy [32], to calibrate and monitor medical devices that use radiation [35], to control the quality of industrial products such as construction materials and food products [39], for radiation detection in non-destructive testing of industrial materials [19], in radiometric dating and analysis of the isotopic composition of geological and archaeological samples [31], for chemical analysis of samples containing radioactive isotopes [27], to monitor radiation levels in nuclear reactors and to analyze the composition of nuclear fuel [20], or to analyze and classify radioactive waste before disposal [36].

Biological radiation dosimeters (biodosimeters) are used to assess exposure to ionizing radiation based on biological changes in living organisms. They are, for example, used to evaluate the radiation doses received by patients during radiation therapy, which helps optimize treatment plans [35], to evaluate radiation exposure in diagnostic procedures such as computed tomography (CT) and other imaging tests [32], to monitor workers’ radiation exposure in high-risk environments such as medical facilities and the nuclear industry [24], to assess doses received by individuals from accidental radiation events and to study the effects of ionizing radiation on cells and organisms, allowing for a better understanding of the mechanisms of radiation damage [44], in epidemiological studies to evaluate the long-term health effects of radiation exposure in various populations [45], to assess the impact of radiative emissions from nuclear power plants on the nearby environment and population [20], to assess the potential radiation effects of radioactive waste on biological monitoring systems [36], in training programs for medical and technical personnel to increase awareness of the biological effects of radiation and radiation protection procedures [46], and in response planning for radiation emergencies, allowing exposure levels to be quickly assessed and appropriate actions taken [47].

Finally, a general comparative analysis is presented in Table 1 describing key advantages and limitations of each proposed method of detection. The enlisted methods for detecting ionizing radiation present unique upsides and disadvantages, making them suitable for specific applications. The devices, based on Geiger–Müller counters, are simple and robust, and by providing real-time measurements are ideal for field use and safety monitoring. When it comes to solid-state detectors, the offered high precision and sensitivity make them essential for medical imaging and research. The scintillation detectors can be described as versatile and sensitive, and are encountered in numerous devices made for medical and environmental monitoring. Thermoluminescence detectors are excellent for passive detection and particularly suited for long-term dose measurement. The photocolorimetric methods, based on the color change in an active chemical, provide a simple, cost-effective visual indication of radiation presence. Biological methods are the only method allowing assessment of the direct biological effects of radiation on living organisms and the environment. The choice of detection method should depend on many factors important for the final application, such as measurement sensitivity and accuracy, operational conditions, size and shape of the device, and the ionization-type radiation expected for measurements [48,49,50]. 

### 2.2. LCD Technologies and Their Potential for the Detection and Readout of IoR

The revolution in display technologies came with the discovery of useful, birefractive structures of mesophases—intermediate states of matter exhibited with organic materials known as liquid crystals—LCs [51]. Commonly applied LCs comprise organic molecules of a strongly anisotropic, mostly elongated shape. At the mesogenic state, they exhibit the preferred direction of the orientation of long molecular axes marked with a unit vector—the so-called molecular director. Such a structure implies that LCs are characterized by electric permittivity anisotropy and optical birefringence. Anisotropic LC structures, especially structurally uniform thin layers formed between transparent substrates, respond to the external electric field action. An electric field generated between transparent electrodes drives the reorientation of the molecular director hence the reorientation of the optical axis of the medium. This way, a thin, electric field-driven LC slab, placed between polarizers, can modulate the transmission of the light incident on the whole structure. Such an effect is commonly used in ubiquitous liquid crystal displays (LCDs) [52] to generate modulated intensities of red, green, and blue pixels when set up in arrayed structures. Considerable attention has been given to a specific type of LC structure, so-called twisted nematics (TNs). Such a structure is formed by the LC comprised of homochiral molecules (at least in a certain amount of dopant) when it is placed between two separated flat substrates, inducing a mutually orthogonal direction of the molecular director. In a cell, the molecular director adiabatically rotates by 90° when observed from substrate to substrate. The other version of such a structure, called a super twisted nematic (STN), is obtained when the LC structure forms a 180° twist. When a TN slab of a proper thickness (fitting so-called Mauguin regime) is placed between two crossed polarizers, it rotates the polarization plane of light which passed the first polarizer by exactly 90°, allowing it for unaffected passing through the second polarizer. The untwisting process within the TN slab, modulated by the electric field intensity, alters the light passing through the whole transducer [53]. Such a process is characterized with a threshold voltage $V_{th}$, dependent mainly on the LC elastic constants, LC electric permittivity anisotropy, and the LC slab thickness. Taking advantage of a threshold and adiabatic switching, the TN structure is considered for application for the IoR converter.

### 2.3. Current Solutions and Vision of the Future

**Figure 2:**
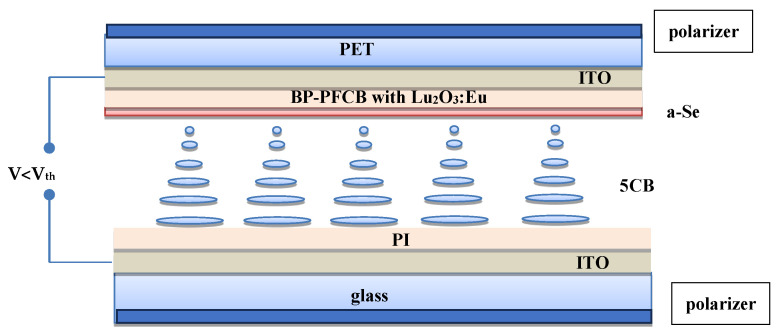
The cross-section of CC with all functional layers indicated. ITO is a transparent electrode, BP-PFCB (biphenylperfluorocyclobutyl) dopped with Lu_2_O_3_:Eu nanoparticles, α-Se is a selenium layer; 5CB is 4-cyano-4-pentylbiphenyl—a nematic liquid crystal doped with some amount of a chiral dopant, forming a TN structure; PI is unidirectionally rubbed SE130 polyimide for LC ordering.

The cascade converter (CC) according to the proposed solution, is a compact element comprising a three-layer structure (see Figure 2) for the cascade conversion of IoR to the naked-eye visible picture. The first layer of the transducer converts IoR into non-ionizing electromagnetic radiation, i.e., at visible range. The second conversion stage converts this radiation into an electric charge distributed over the surface. The third conversion stage converts the distributed electric charge into the distribution of the electric field driving the liquid crystal TN cell. Depending on the generated potential, the degree of deformation of the nematic structures changes. The effect of this phenomenon is an observable change in the transparency of the liquid crystal-filled transducer. The change in radiation intensity is therefore proportional to the change in the transparency of the indicating CC. 

The first conversion stage in the proposed CC is a radioluminescent layer made of Lu_2_O_3_:Eu nanocrystals. The second stage of conversion is created by a layer of photoconductive material, here amorphous selenium α-Se. 

**Figure 3:**
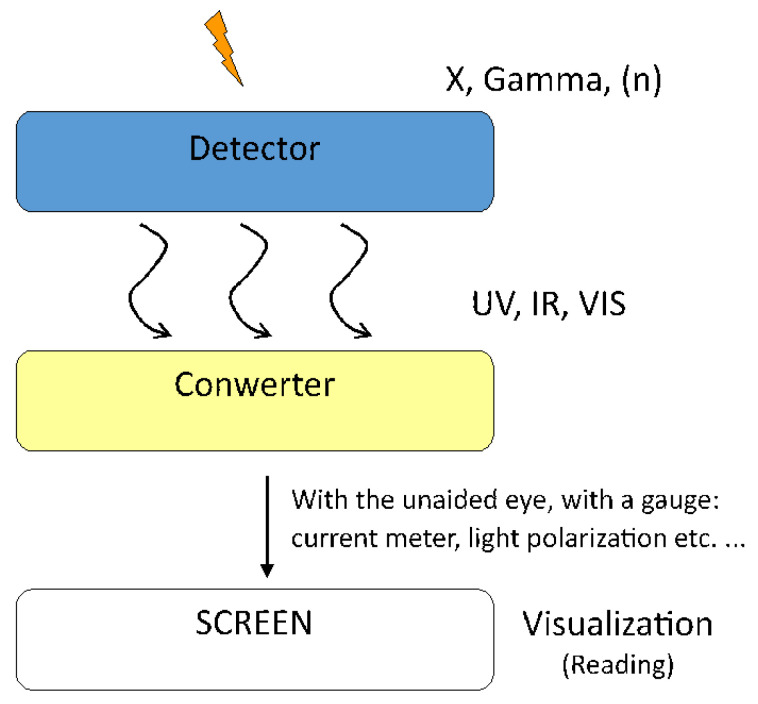
Scheme of three conversion stages in CC.

The TN cell serves as a visualizing element that changes its transparency depending eventually on the intensity of the primary factor, which is IoR (Figure 3).

The proposed CC circumvents a serious limitation of the aforementioned IoR detectors and measuring tools, which is the complex structure and a need for high-voltage power supply. Here, the IoR energy is converted into electric charges which finally affect the magnitude of the electric field driving TN transparency.

## 3. Experimental Part

### 3.1. Materials and Methods

4-Cyano-4-pentylbiphenyl (5CB) was purchased from Sigma-Aldrich. SE 130 polyimide (PI) for a planar LC orientation was purchased from Nissan Chem. Glass with transparent electrodes of the indium—tin—oxide (ITO) and PET/ITO are commercially available. α-Se with a thickness of c.a. 100 nm was deposited by vacuum evaporation based on our previous experience [54]: the base pressure was maintained at ~10^−6^ Torr with the temperature of molybdenum boat heated at ~250 °C and applying large AC (100–150 A).

**Figure 4:**
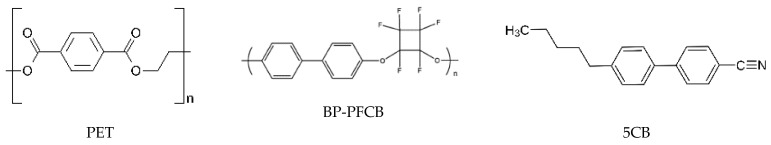
Molecular structures of polymer materials used as well as the liquid crystalline compound 5CB.

Biphenylperfluorocyclobutyl polymer (BP-PFCB) was purchased from Tetramer Technologies, Mechanic ST, Pendleton, SC, USA. The molecular structures of polymer materials used as well as the liquid crystalline material 5CB are presented in Figure 4.

Europium-doped Lu_2_O_3_:Eu nanocrystalline powders were prepared via a combustion route as presented in our previous work [55]. The morphology of the sample was analyzed using a Hitachi S-3400N scanning electron microscope (Hitachi High-Technologies, Tokyo, Japan) and a FEI Tecnai G2 20 X-TWIN transmission electron microscope (FEI, Eindhoven, The Netherlands). Photoluminescence (PL) and PL excitation spectra were recorded with an FLS1000 Fluorescence Spectrometer from Edinburgh Instruments, Ltd. (Livingston, UK). A 450 W xenon arc lamp was used as the excitation source. TMS302-X double grating monochromators of 325 mm focal lengths were used, and the luminescence spectrum was recorded with a Hamamatsu R928P high-gain photomultiplier detector (Herrsching, Germany), thermoelectrically cooled to −20 °C. The excitation spectra were corrected for the incident light intensity and the emission spectra were corrected for the spectral sensitivity of the recording system.

The performance of the prepared CC was valued when it was irradiated with X-rays using the URK X-ray lamp of the diffractometer TUR M53 with Cu anode without filters. X-rays were generated at two different intensities obtained when the source was supplied with 15 mA and 20 mA, both at 20 kV. The CC was placed c.a. 28 cm off the lamp window. The converter plane was oriented near perpendicular to the X-ray beam. 

**Figure 5:**
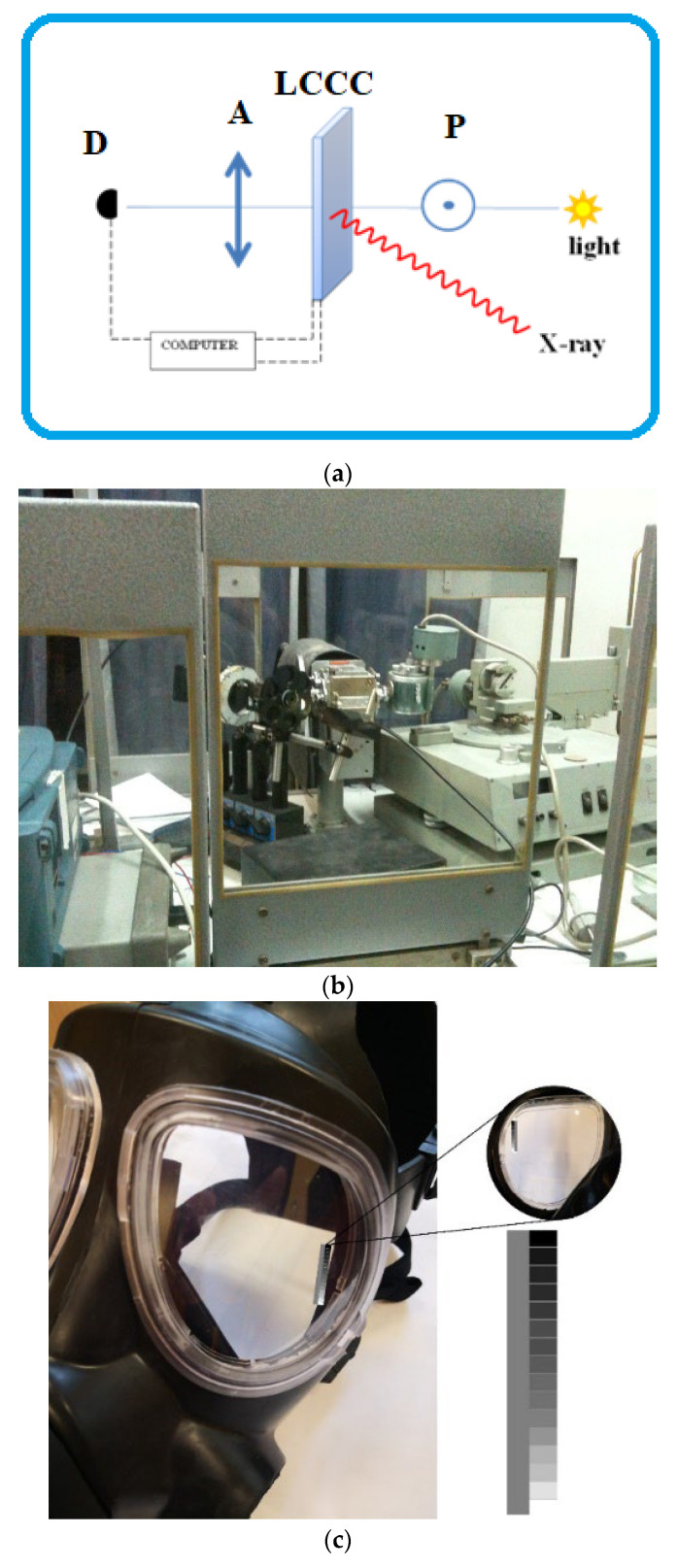
(**a**) A scheme of the measuring set-up. D—detector (FLCE PIN 20 photodiode), A—analyzer, P—polarizer. (**b**) Photo of measuring set-up with diffractometer TUR M53 with URK X-ray source with Cu anode (right site). (**c**) Photo of constructed device.

To measure the CC response for the X-ray (actually observed with the naked eye), the halogen lamp illuminating the CC and linear detector FLCE PIN 20 of the transmitted light intensity, properly shielded against X-rays, were installed. The schematic representation of the measurement system is presented in Figure 5 along with a photo of the experimental set-up and constructed device. 

### 3.2. Aspects of Selected Materials 

Designing a straightforward IoR converter structure that enables the detection and reading of the radiation distribution is a complex task. A final effect is dependent mainly on the sensitivity and performance of the functional components used. Using LCD-like, TN technology for imaging, as well as proper absorbers and converters for sensing, the IoR-driven CC is feasible to design and fabricate. Proper tailoring of all functional layers of the converter allows detection and observation of the intensity of the IoRs depicted with the TN cell’s gray levels. At this mode of operation, the converter could be considered as an effective individual detector showing online the distribution of IoR’s energy density and even as a radiometer when the readout is integrated over time. The particularities of the converter elements and their role are discussed below. 

To ensure a minimal attenuation of the IoR incident, the sensing part of the converter on one of the substrates of the testing device was made of a transparent polymer plate (here poly(ethylene terephthalate)—PET) with a thickness of 0.2 mm. Such a substrate was equipped with a transparent electrode made of ITO with a specific resistivity of 60 Ω/sq. The substrate was thin enough to radically reduce the IoR absorption in comparison with a typical borosilicate display glass substrate of a thickness as low as 0.5 mm. As an essential element of a CC, a thin sensing layer of a polymer doped with functional nanoparticles (NPs) was used. Here a polymer used was biphenyl perfluorocyclobutyl—BP-PFCB [56,57]. BP-PFCB forms a proton exchange semipermeable membrane which supports charge separation. BP-PFCB doped with Lu_2_O_3_:Eu functional nanoparticles (NPs) was deposited over the ITO transparent electrode by spin-coating of the solution in cyclohexane. The thickness of NPs-doped BP-PFCB film was c.a. 100 nm. The obtained IoR sensing layer is thermally stable at the CC’s working temperature ranges and the long-time storing is durable and transparent. The used luminophore was europium-doped Lu_2_O_3_ nanoparticles. It was doped at the concentration of 12% by weight of Lu_2_O_3_:Eu in BP-PFCB. The average diameter of NPs used was 11 nm, according to the XRD studies [58]. We chose this compound due to their operation at the IoR conversion, where:

(i) luminescence: Lu_2_O_3_:Eu exhibits an intensive red light emission when excited with UV or shorter wavelength radiation [59,60];
(ii) high quantum efficiency exhibited by Lu_2_O_3_:Eu ensures a high yield of radiation emitted at the VIS range [61,62], moreover, the luminescence of Lu_2_O_3_:Eu can be stimulated by UV radiation at the appropriate wavelength [63,64];
(iii) the high thermal stability of Lu_2_O_3_:Eu makes it resistant to high temperatures compared to alternative converters like red phosphors [65,66];
(iv) durability: the luminescence of Lu_2_O_3_:Eu is durable and does not degrade with time and irradiation intensity.

**Figure 6:**
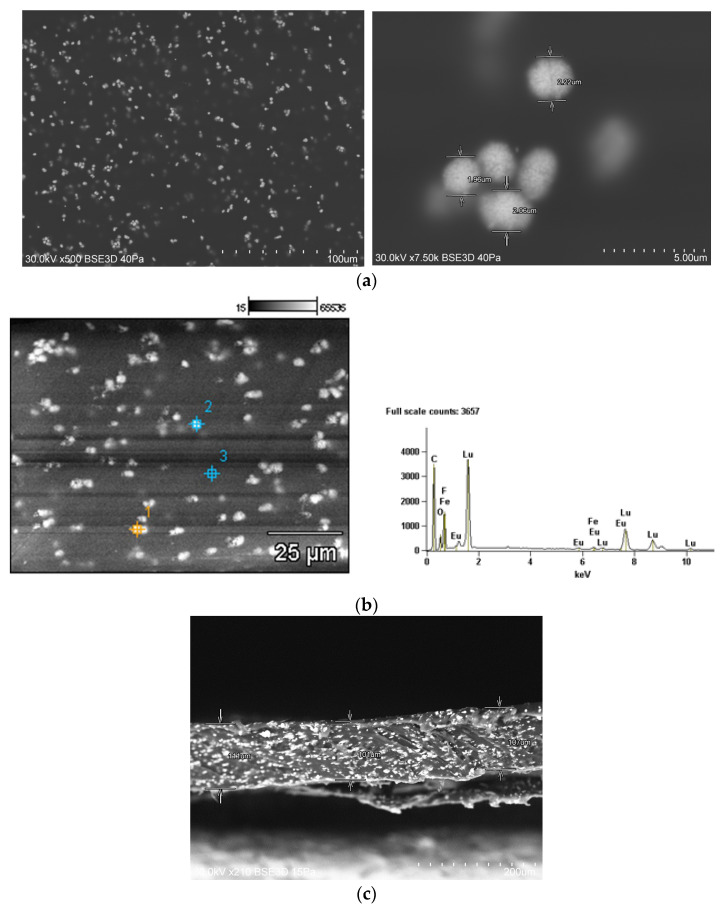
TEM (**a**) and SEM images and (**b**) energy dispersive X-ray spectroscopy (EDS) data of Lu_2_O_3_:Eu. (**c**) SEM photo displays a cross-section of the slab with a thickness of c.a. 100 µm of BP-PFCB polymer doped with Lu_2_O_3_:Eu NPs.

The morphology of Lu_2_O_3_:Eu was determined with TEM and SEM measurements (see Figure 6a,b). The high-resolution TEM images (Figure 6a) show the spherical uniform size of the luminophore particles whose diameters were about 200 nm. EDS analysis confirmed that Eu was effectively incorporated into the host lattice (Figure 6b). 

**Figure 7:**
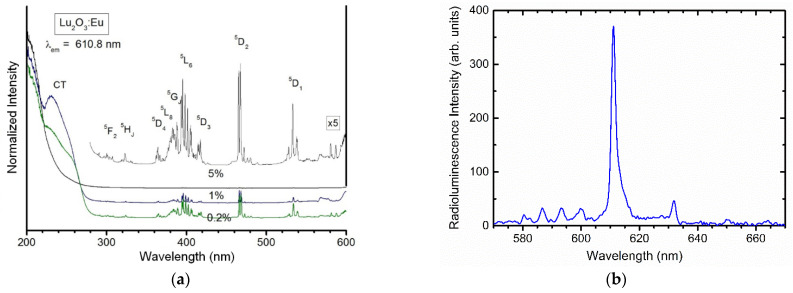
(**a**) Excitation spectra of Lu_2_O_3_:Eu NPs of the 610.8 nm emission measured at room temperature for three different concentrations of Eu^3+^ ions. (**b**) Observations of the radioluminescence of BP-PFCB doped with Lu_2_O_3_:Eu. The layers’ thicknesses were approx. 100 µm. Maximum emission was observed at 610.8 nm.

Excitation spectra of the emission of Lu_2_O_3_:Eu at 610.8 nm were measured at room temperature for three different concentrations of Eu^3+^ ions in NPs: 0.2, 1, and 5%—see Figure 7a. Obtained photoluminescence spectra are characteristic for Eu^3+^ luminescence with a dominating charge-transfer broad-band transition around 250–270 nm. Using synchrotron radiation, the fundamental absorption of the host was found to appear below 220 nm [67]. The band seen in this range in the photoluminescence spectra confirms the efficient energy transfer from the host to the activator which finally generates its red luminescence, as expected in high-quality X-ray luminophore.

Finally, we investigated the luminescence of BP-PFCB with Lu_2_O_3_:Eu in a layer of approximately 100 µm. The radioluminescence spectrum (Figure 7b) confirms that the polymer-NP composite described above easily generates the “red photons” upon exposure to X-rays, and the main peak at 610.8 nm results from the ^5^D_0_→^7^F_2_ transition of Eu^3+^ ions.

BP-PFCB semipermeable film containing the luminescent NPs was then covered with a layer of a photoinduced charge generator—α-Se, the most popular material for photoconductivity observations. The layer thickness of metallic α-Se was c.a. 100 nm. The α-Se layer was thin enough to maintain the transparency of the IoR converter. Properties of α-Se most important for the LC CC performance and durability are:

(i) photoelectric effect: α-Se exhibits a high electron–hole pair extraction efficiency when affected by radiation at the visible range, which makes it an ideal material for use in the fabrication of photovoltaic cells [68]; moreover, α-Se remains the only amorphous photoconductor where while drifting in an electric field, charges (here, holes are of higher charge mobility than for electrons) can avoid energy dissipation and hence can acquire enough energy to initiate impact ionization and secondary charge creation;
(ii) light absorption: α-Se absorbs the electromagnetic radiation at VIS and IR ranges [69];
(iii) X-ray sensitivity: α-Se has high X-ray sensitivity, making it an ideal material for applications in medical imaging [70];
(iv) thermal stability: α-Se has high thermal stability, which means that it can be used where high operating temperatures are required [71,72];
(v) electrical properties: α-Se also has unique electrical properties, like a low current activation temperature, which means it can function at lower voltages than traditional semiconductor materials [73].

To ensure the proper alignment of the optically active LC slab, the α-Se layer was spin-coated with a solution of polyimide (PI). It was subsequently dried at 80 °C and cured at 180 °C for half an hour. Next, a rubbing process of the PI layer was conducted to induce effective orienting properties of PI. Here the PI layer properties and rubbing procedure were optimized for inducing a TN structure of 5CB. The 5CB LC was doped with a chiral dopant (CD) as to avoid random twisting domains induced within a converter gap [74,75]. 

The opposite substrate of the IoR converter was made of float-type glass used routinely for LCD technology, where the ITO electrode was covered with another PI layer rubbed in the direction perpendicular to the one described above. Both substrates were assembled with glass rods (diameter of 5.0 ± 0.01 μm) as spacers deposited over the IoR converter substrate. Spacers ensured the uniform gap between CC substrates. The gap was filled with 5CB liquid crystals at the isotropic state (at elevated temperature) with capillary action. After cooling to the room temperature, 5CB formed at a twisted structure (TN). ITO layers at both substrates were wired to provide the electric potential to the electrodes, hence inducing an electric field within the CC. The IoR converter was placed between crossed polarizers and was optically inspected while driving with voltage applied to the transparent electrodes. In our case, the test TN transducer worked in the “normal white” mode.

Analyzing the mechanism of IoR-driven generation of the potential difference between ITO and α-Se layers, which affects switching of the TN structure, one can conclude that the value of the potential difference was approx. 1.2–1.8 V with a negative potential on the selenium surface. Therefore, with the BIAS polarization between opposite ITO electrodes, the IoR-induced additional potential difference drives the TN structure to switch. 

## 4. Results and Discussion

### Selected Opto-Electrical Parameters of Cascade Converter

**Figure 8:**
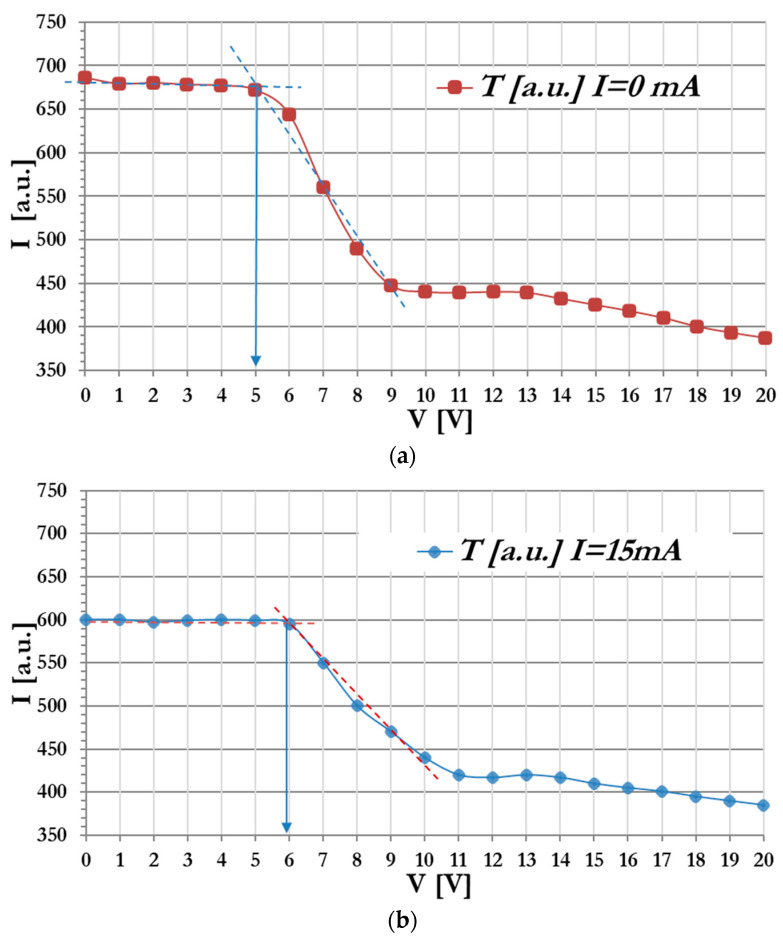

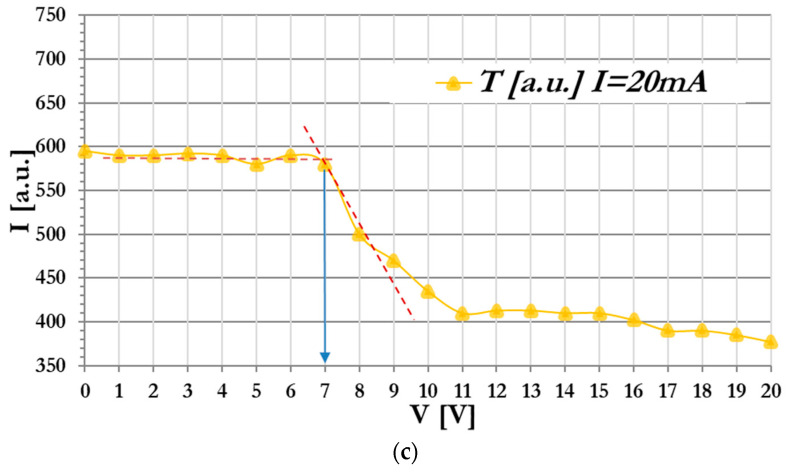
Light intensity I (in arbitral units) passing through the CC vs. the value of the driving voltage V: (**a**) without the influence of X radiation (threshold voltage V_0_ = 5.0 V), and at the X-ray generated at a current of (**b**) 15 mA with the threshold voltage shifted to V_0_ = 5.9 V, and at current (**c**) 20 mA with the threshold voltage shifted to V_0_ = 6.9 V. Dashed lines were used for the evaluation of the threshold voltage.

The observations of electro-optical characteristics of the CC with the asymmetrical cell with PET (100 µm)/ITO (50–100 nm)/BP-PFCB doped with Lu_2_O_3_:Eu (100 nm)/α-Se (100 nm)/5CB/PI (50–100 nm)/ITO (50–100 nm)/glass architecture were performed for two X-ray intensities obtained at 15 mA and 20 mA currents applied to a Cu lamp emitting X-rays. Under the illumination of the CC with X-rays, an increase in the TN threshold switching voltage was observed, namely, changing from 5.0 V to 6.0 V and 7.0 V, respectively, for different X-ray intensities (see Figure 8).

We observed a significant increase in the value of the threshold voltage from $V_{th}=5.1\;V$ (at no X-ray action) to $V_{th}=6.9\;V$ (at X-ray intensity obtained at the current of the Cu cathode of 20 mA). The change in the TN switching threshold voltage $V_{th}$ by 1.8 V results with the conversion of IoR (here X radiation) into the electro-optical effect observed in the CC. Therefore, it is possible to observe changes in the transparency of the CC cell depending on the attenuation of radiation passing through various “obstacles”, i.e., human tissue, building structures, dangerous objects hidden in luggage, etc. The change in the transparency of the CC cell/pixel, from transparent to dark state, will occur in real time and will be visible to the naked eye.

## 5. Conclusions

Thanks to the use of multi-stage conversion, the CC is characterized by:

⮚ Construction based on three layers only, which has a beneficial effect on minimizing both the time and costs of converter production (technological aspects) and is an advantage of the proposed CC.
⮚ The CC, according to the proposed technology, registers lower radiation energies than other traditionally used measuring devices.

The constructed X-ray and gamma-ray CC can be used to detect dose-equivalent fluctuations in real time.

## Figures and Tables

**Table 1:** Comparative brief of methods used to detect and/or measure IoR.

Methods	Advantages	Limitations	Ref.
Gas ionizing radiation counters	- simple and robust construction, i.e., the Geiger–Müller counters are simple to use and rugged, making them ideal for field applications; - detect a wide range of radiation, namely, alpha, beta, and gamma radiation, depending on used window material and gas; - able to monitor in real time, giving immediate feedback on radiation levels.	- less precise with lower energy resolutions, typically less precise in distinguishing different radiation energies compared to solid-state detectors; - Geiger–Müller counters have limited sensitivity at low levels of radiation and may not measure low-intensity radiation accurately; - have ‘Dead Time’ period, a time required to work accurately between two measurements; moreover, affect measurement rates at high radiation levels.	[48]
Solid ionizing radiation counters	- are characterized by high-energy resolution, which means are able to distinguish different types and energies of radiation with high precision; - in terms of size are small and portable, making them suitable for a variety of applications; - are characterized by high sensitivity enabling effective detection of low-level and high-intensity radiation.	- due to their thermal sensitivity, the performance can be affected by the temperature. Cooling systems are required to overcome this issue in high-precision applications; - in general, are more expensive due to the complexity and type of used materials; - prone to damage after prolonged exposure to high radiation levels.	[48]
Scintillation radiation detectors	- due to their sensitivity are suitable for detecting low-level radiation and various radiation; - thanks to good energy resolution, particularly suitable for gamma spectroscopy; - since they are available in numerous sizes and forms, can be used in many applications, i.e., portable handheld devices.	- are sensitive to moisture (hygroscopic nature), and require protective encapsulation; - the ability to generate light can decline over time, especially when exposed to high radiation; - for some applications, scintillator-based detectors would require considerable size and bulkiness.	[48]
The thermo-luminescence (TL) method	- is able to measure a cumulative radiation in a passive way over time without external power source; - allows construction of dosimeters with wide dose range; - structural stability and reusability after annealing, making them very cost-effective for long-term applications.	- does not provide real-time reading, only information about cumulative dose exposure; - are sensitive to external conditions, such as heat and light; - requires specific equipment for the visualization of accumulated dose reading.	[48]
The photo-colorimetric method (PC)	- allows direct and simple visualization of radiation exposure; - is based on relatively inexpensive materials and is easy to use; - its operational principle is not based on an external power source.	- offers limited accuracy in comparison to electronic detectors; - the visual indication can be affected by some environmental conditions, such as light, temperature, or humidity; - operates mainly in limited radiation dose range.	[49]
The biological method (BioM)	- allows direct measurement of irradiation dose effect on biological systems; - suitable for study of adiation effects on living organisms and ecosystems; - is called a natural dosimeter due to use of organisms and systems inherently sensitive to radiation and present in natural ecosystems.	- frequently require sophisticated biological assays and extended analysis times, hence complex and time consuming; - allows observation of qualitative effects of irradiation, therefore less precise when compared to physical detectors; - use of living organisms affect the ethical and practical aspects of this method.	[50]

## Data Availability

The authors declare that the data supporting the findings of this study are available within the paper.
